# Complete Genome Sequence of Hyperthermophilic Archaeon *Thermococcus* sp. EXT12c, Isolated from the East Pacific Rise 9°N

**DOI:** 10.1128/genomeA.01385-17

**Published:** 2017-12-14

**Authors:** Damien Courtine, Karine Alain, Myriam Georges, Nadège Bienvenu, Hilary G. Morrison, A. Murat Eren, Loïs Maignien

**Affiliations:** a Université de Bretagne Occidentale (UBO, UBL), Institut Universitaire Européen de la Mer (IUEM), UMR 6197, Laboratoire de Microbiologie des Environnements Extrêmes (LM2E), Plouzané, France; b CNRS, IUEM–UMR 6197, Laboratoire de Microbiologie des Environnements Extrêmes (LM2E), Plouzané, France; c Ifremer, UMR 6197, Laboratoire de Microbiologie des Environnements Extrêmes (LM2E), Plouzané, France; d Josephine Bay Paul Center for Comparative Molecular Biology and Evolution, Marine Biological Laboratory, Woods Hole, Massachusetts, USA; e Department of Medicine, University of Chicago, Chicago, Illinois, USA

## Abstract

We report the genome sequence of *Thermococcus* sp. EXT12c isolated from a deep-sea hydrothermal vent at the East Pacific Rise 9°N. Microbes in the genus *Thermococcus* are able to grow anaerobically at high temperature, around neutral pH, and some of them under high hydrostatic pressure.

## GENOME ANNOUNCEMENT

We isolated *Thermococcus* sp. EXT12c from a hydrothermal chimney rock sample collected from a 2496-m depth near the East Pacific Rise 9°N (9°50′40.2″N, 104°17′37.798″W) during the oceanographic cruise EXTREME (October 2001). *T.* sp. EXT12c is able to grow under anaerobic conditions, at 85°C and pH 6.8 in TRM medium (1). The strain is available in the UBOCC culture collection (Brest, France) under the reference no. UBOCC-M-2417.

We used a phenol-chloroform technique for DNA extraction and the TruSeq DNA PCR-free kit (Illumina, USA) to prepare paired-end sequencing libraries with an average insert size of 550 nt. Whole-genome sequencing at the Marine Biological Laboratory (Woods Hole, MA, USA) using an Illumina MiSeq machine (MiSeq reagent kit v3) produced 1,335,523 2 × 300 bp reads after quality filtering (2). Our *de novo* assembly with CLC Genomics Workbench v8.5.1 (https://www.qiagenbioinformatics.com/products/clc-genomics-workbench) resulted in a single chromosome with 2,155,760 nt and a GC content of 54.58%. This single chromosome recruited 98.8% of the short reads, with an average coverage of 350×.

The MaGe genome annotation platform (3–14) identified 2,365 coding sequences; a single 16S-23S operon; and 2 5S rRNA, 46 tRNA, and 16 miscellaneous RNA genes. InterProScan identified 1 integrase, 6 transposases, and 3 clustered regularly interspaced short palindromic repeat (CRISPR) loci associated with *cas* genes (*cas*, *cst*, and *cmr*), suggesting that the strain probably carries two types of CRISPR systems, class I type I and type III (15). These features suggest that the strain has a certain genomic plasticity.

Among the *Thermococcus* species with published genomes, *T*. sp. EXT12c is most closely related to *T. nautili* strain 30-1^T^ (16). These genomes have a DNA-DNA hybridization value of 43.6% and an average nucleotide identity of 91.18%, as predicted with GGDC v2.1 (17–19) and OrthoANI v1.20 (20), respectively.

*T.* sp. EXT12c possesses some complete metabolic pathways like the glycolysis and amino acid biosynthesis pathways for alanine, asparagine, glycine, glutamate, and tryptophan. To date, only ten *Thermococcales*, including *T. kodakaraensis*, *T. litoralis*, *Pyrococcus furiosus*, and *P. abyssi* (21–25), harbor a complete tryptophan biosynthesis pathway.

In this metabolic pathway, a single locus contains genes leading to the synthesis of chorismate, an intermediate for multiple metabolic pathways (TEXT12C_2159 to TEXT12C_2167), and tryptophan (TEXT12C_2174 to TEXT12C_2168, genes *trpCDEGFBA*). Within the *T. kodakaraensis* genome, genes that code for tryptophan biosynthesis are present in a single locus too (26). A transcriptomic study showed that the chorismate synthesis is downregulated when *T. kodakaraensis* is cultivated under high hydrostatic pressure, compared to atmospheric pressure (27). However, in the same study, the gene *trpC*, labeled TK0252 in *T. kodakaraensis*, is upregulated, indicating that the strain could continue to produce tryptophan and compensate the decrease of chorismate production under high pressure. Therefore, the regulation of tryptophan biosynthesis in *T*. sp. EXT12c under high-pressure conditions requires further investigations.

### Accession number(s).

This genome sequence has been deposited in DDBJ/ENA/GenBank under the accession no. LT900021. The version described in this paper is the first version.
